# Integration of Vulnerability and Hazard Factors for Landslide Risk Assessment

**DOI:** 10.3390/ijerph182211987

**Published:** 2021-11-15

**Authors:** Patricia Arrogante-Funes, Adrián G. Bruzón, Fátima Arrogante-Funes, Rocío N. Ramos-Bernal, René Vázquez-Jiménez

**Affiliations:** 1 Department of Chemical and Environmental Technology, ESCET, Rey Juan Carlos University, C/Tulipán s/n, 28933 Móstoles, Madrid, Spain; 2 Departamento de Geografía, Geología y Medio Ambiente, Facultad de Filosofía y Letras, Universidad de Alcalá, Área de Geografía, GITA, C/Colegios 2, 28801 Alcalá de Henares, Madrid, Spain; fatima.arrogante@uah.es; 3 Cuerpo Académico UAGro CA-93 Riesgos Naturales y Geotecnología, FI, Universidad Autónoma de Guerrero, Av/Lázaro Cárdenas s/n, CU, Chilpancingo 39070, Mexico; rnramos@uagro.mx (R.N.R.-B.); rvazquez@uagro.mx (R.V.-J.); 4 Research Group on Technologies for Landscape Analysis and Diagnosis (TADAT), Department of Chemical and Environmental Technology, ESCET, Rey Juan Carlos University, C/Tulipán s/n, 28933 Móstoles, Madrid, Spain

**Keywords:** landslide, hazard assessment, landslide risk, vulnerability, susceptibility, ecological values

## Abstract

Among the numerous natural hazards, landslides are one of the greatest, as they can cause enormous loss of life and property, and affect the natural ecosystem and their services. Landslides are disasters that cause damage to anthropic activities and innumerable loss of human life, globally. The landslide risk assessed by the integration of susceptibility and vulnerability maps has recently become a manner of studying sites prone to landslide events and managing these regions well. Developing countries, where the impact of landslides is frequent, need risk assessment tools that enable them to address these disasters, starting with their prevention, with free spatial data and appropriate models. Our study shows a heuristic risk model by integrating a susceptibility map made by AutoML and a vulnerability one that is made considering ecological vulnerability and socio-economic vulnerability. The input data used in the State of Guerrero (México) approach uses spatial data, such as remote sensing, or official Mexican databases. This aspect makes this work adaptable to other parts of the world because the cost is low, and the frequency adaptation is high. Our results show a great difference between the distribution of vulnerability and susceptibility zones in the study area, and even between the socio-economic and ecological vulnerabilities. For instance, the highest ecological vulnerability is in the mountainous zone in Guerrero, and the highest socio-economic vulnerability values are found around settlements and roads. Therefore, the final risk assessment map is an integrated index that considers susceptibility and vulnerability and would be a good first attempt to challenge landslide disasters.

## 1. Introduction

Among the numerous natural hazards, landslides are one of the greatest, as they can cause enormous loss of life and property, and affect the natural ecosystem and their services [1]. Landslides are disasters that cause damage to anthropic activities and innumerable loss of human life, globally [2]. Landslide events constitute 5% of natural disasters, globally. This latter comment supposes high risks, because landslides are associated with other natural disasters such as earthquakes, eruptions, hurricanes [3]. In the last decades, this natural risk has increased, on a global scale, due to urbanization and, in general, urban growth in areas with a high occurrence of landslides, and due to deforestation and increased regional or local rainfall caused by global change [4,5]. These extreme natural events cannot be foreseen, but their risk can be reduced by taking precautions and security and alarm measures. Therefore, it is important to know, through indicators, a tangible and interpretable value of the risk they pose [6,7]. To reduce and mitigate the risk allied with this natural phenomenon, processes of detecting and understanding the landslides causes must be developed, to encourage prevention policies, early warning systems and recovery programs [8].

We define risk as the combination of the physical probability that an event occurs (susceptibility) and the potential damage that this event could generate (vulnerability) [9]. Following the latter commented definition, we must place the evaluation of the landslide risk into a framework that combines these two concepts, susceptibility (as a proxy of hazard) and vulnerability. The most appropriate way to challenge the landslide event in recent decades is the spatial assessment of susceptibility and vulnerability to landslides [10,11]. The occurrence of landslides is a natural phenomenon, but anthropogenic processes are one of the causes of vulnerability to landslides. This fact makes it difficult to predict landslides’ spatial and temporal occurrence [12]. In this sense, landslide susceptibility and vulnerability maps must be used as the main method to classify the high and low risk areas that are prone to landslides and, thereby, they can help in finding the variables responsible for the existence of landslides [13,14]. With the development of different tools, hardware, and the availability of spatial and even multitemporal data, it has become easier than in the last century to produce landslide susceptibility and vulnerability maps [15].

In landslide spatial studies, often, susceptibility is related to causes intrinsic to physical predisposition (for instance: topographical variables, climatic, etc.), and vulnerability usually corresponds to external influences along with causes intrinsic to the physical (anthropic exposure) [16]. The concept of vulnerability is defined in natural hazards terminology as: “The characteristics and circumstances of a community, system or asset that make it susceptible to the damaging effects of a hazard” [17]. Previous landslides studies have not considered the vulnerability aspect, while they are based on susceptibility modelling. Therefore, in the present approach, the novelty is the overall assessment of vulnerability. Given that the level of vulnerability is negatively associated with economic and technological development, the creation of vulnerability indicators should be a priority for international disaster reduction programs [18].

In the vulnerability assessment, on the one hand, socio-economic factors must be considered. On the other, we must consider the ecological landscape factors; finally, all of them must be integrated. Unfortunately, there is no standard methodology to evaluate vulnerability at various locations under different hazard levels [19]. However, landslide vulnerability is vital for effective landslide disaster risk reduction to minimize the damage of lives and property [20]. In the bibliography regarding the study of vulnerability to landslides, some articles have addressed socio-economic vulnerability, including marginality, population, state of buildings, and income, among others [10]. However, carrying out vulnerability assessments that include ecological and landscape vulnerability, which also consider the delay in the regeneration of ecosystems, is not widely studied. In this sense, our approach will address both socio-economic vulnerability and ecological landscape vulnerability, understanding this latter as the inability of an ecosystem (structural and functional component) to tolerate temporal and spatial pressures [21].

The integration of RS-GIS techniques with knowledge driven methods or data-mining methodologies in landslide susceptibility and vulnerability assessment, using spatial and nonspatial data, has been largely used [22]. Multicriteria decision and machine learning models, in susceptibility studies, bring good results [14]. The ML methods for susceptibility assessment studios provided better results than traditional approaches because nonlinear data can be satisfactorily handled by ML models with various scales [23]. Thereby, we use a susceptibility map made through an ML model [24]. For vulnerability, we present a method based on the combination of ecological landscape vulnerability and a socio-economic heuristic method, in which we combine free data sources with continuous spatial coverage. This methodology will be implemented in the State of Guerrero, Mexico. Still, it would be applicable anywhere on the planet precisely because of the search for free and easily updated sources, since they will be available by remote sensing, for the most part.

The present paper will develop a conceptual framework and a prototype based on remote sensing and GIS databases (overall free) to assess the risk of landslides based on the integration of susceptibility and vulnerability through ML and heuristic methods. This product may facilitate a better understanding of the future impacts of landslide events in the State of Guerrero in México and offer a low cost methodology to efficiently make emergency plans in this zone in which the landslide events are present and cause high losses.

## 2. Materials and Methods

**Figure 1:**
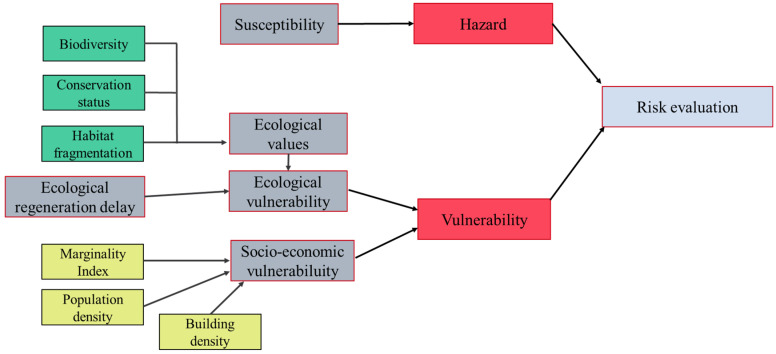
Components of landslide risk assessment.

Figure 1 includes a schematic presentation of the components of landslide risk, made by integrating both landslide susceptibility and vulnerability. The methodology used to calculate the landslide risk and the cartography is based on [8] and [25], and adapted for the authors of the present work. Although our methodology has been proposed for Guerrero in México, it is a universal methodology that can be extended to any part of the world. 

As seen in the present Section 2 (Materials and Methods), the scheme was developed around two components: susceptibility and vulnerability. Susceptibility was proposed by the authors in [24]. In the aforementioned article, the authors presented a methodology based on an automatic machine learning (AutoML) framework, comparing the performance of 16 machine learning algorithms in the center and southern region of Guerrero (Mexico). The best results were obtained, and therefore the final susceptibility map was made, with the extra trees model. Landslide inventory was used to train and validate the machine learning models [24]. Besides, to obtain the susceptibility map, we used twelve conditioning factors and one trigger factor. However, the present work covered the overall calculation of landslide vulnerability and the final integration of the risk map. For the landslide vulnerability, we focus on the ecological values (ecological vulnerability) and the human values (socio-economic vulnerability).

On the one hand, the landslide ecological vulnerability was integrated by biodiversity, state of conservation, habitat fragmentation, and ecological regeneration delay. On the other hand, the socio-economic landslide vulnerability was generated by the marginalization index, the population density, and the building density. Besides, in the case of the socio-economic vulnerability, we excluded areas with low landslide probability.

### 2.1. Study Area

The study covers the center and southern region of Guerrero (Mexico). Guerrero is one of the 32 federal states of Mexico, spanning 63,596 km^2^. It is divided into seven areas and is made up of 81 municipalities. Guerrero is in the southern part of the Mexican Republic, and its borders are the states of Mexico and Morelos to the north, the state of Puebla and Oaxaca to the east, the state of Michoacán to the west and the Pacific Ocean to the south (https://www.inegi.org.mx/, accessed on 11 September 2021). Thus, our study area corresponds to 61% of the total surface of the state of Guerrero.

The state of Guerrero is crossed from the northwest to the southeast by the Sierra Madre del Sur [26]. In addition, this state encompasses the Xolapa and Guerrero tectonostratigraphic complex, which are in tectonic contact. Xolapa’s tectonostratigraphic complex has a composition of metamorphic rocks. Guerrero’s tectonostratigraphic complex shows a metamorphosed Vulcan–sedimentary rock composition, and a sequence of metavolcanic sediments and metasediments [27]. Finally, in this state is the Guerrero–Morelos platform, which consists of a series of extensive limestone outcrops deposited in a marine platform environment [28].

The climate of the state of Guerrero is warm subhumid, being temperate subhumid in the mountainous areas. The average annual temperature ranges between 18 and 26 °C, with a relative humidity close to 70% and an average accumulated rainfall of 1106 mm, with the summer months being the wettest [29].

Concerning demography, the state of Guerrero had 3.5 million inhabitants in 2020, 60% of whom live in urban locations, with a density of 56 people per square kilometer, and with the services sector being the main economic activity (76%), followed by the industrial sector (18.4%) (https://www.inegi.org.mx/, accessed on: 11 September 2021). The percentage of the economically active population, those who can work and work or are looking for work, in all municipalities, is below 45%. There are more men than women. The latest commented data means that the other percentages are pensioners or retirees, students, those who do housework or have some limitation that prevents them from working. Several municipalities, such as Alcozauca de Guerrero and Iliatenco, have this percentage below 20%, which indicates a low rate of economically active people. Those with the highest percentage are Zihuatanejo de Azueta and Xochistlahuaca [30]

Concerning ecosystems, coniferous and holm oak forests predominate in the upper parts of the Sierra Madre del Sur, low deciduous forest in the Balsas depression and the Pacific slope, and grasslands, mangroves, and dunes on the fringe. The coast, with agricultural areas, occupies 21% of the state’s surface. Guerrero has ten protected natural areas, of which five are federally legislated and five statewide [30]. A total of 8.4% of the employed population in Guerrero is engaged in commerce, mostly retail, with this being one of the main activities of the state together with nonfinancial private services. Commerce generated 36.2% of the total gross production in the state in 2013. Nonfinancial private services employ 35% of Guerrero’s workforce and account for 29.8% of all gross output [31]. Manufacturing occupies 14.6% of the population of Guerrero, and this is one of the traditional activities of the state: it contributes 11.1% of the total gross production. A total of 4.1% of the working population is dedicated to agriculture, animal husbandry and exploitation, forestry, fishing and hunting, and these activities have a contribution of 4.2% of the total gross production of Guerrero. Mining activities should also be highlighted, because, although only 1% of people work there, it has a large (9.2%) contribution to the state’s total production [31].

Some of the geological risks the study area presents are largely due to volcanism, seismic activity, and the tectonic plates’ activity, at this point. Although Guerrero itself does not have volcanoes, the neighboring states host up to 17, mostly stratovolcanoes, common in subduction plates such as the Cocos plate of the North American plate, an area in which the site is located.

The presence of extraordinary hydrometeorological phenomena has triggered massive landslides that have severely affected the population and infrastructures in recent years. September 2013 was particularly important because of the N.13 tropical depression that occurred in the Pacific Ocean. The subsequent simultaneous hurricanes, Manuel in the Pacific and Ingrid in the Gulf of México, caused significant floods and landslides on the coast of Guererro state. Especially noteworthy is the landslide in the community La Pintada in the municipality of Atoyac, where there were 70 deaths, 379 victims, and 20 damaged buildings [32].

### 2.2. Landslide Danger Assessment

#### 2.2.1. Landslide Inventory

Three different photo interpreters carried out this work on a Google Earth image from 12 August 2014, with the help of GIS software. This date was chosen since the previous year, in September 2013, one of the most active periods of landslides in Guerrero occurred due to the heavy rains that occurred during several consecutive days because of the interaction of the meteorological phenomena Ingrid and Manuel [33]. As a result, the landslide inventory has 518 polygons, or events, of landslides. For our inventory, the precipitation caused by the hurricanes is the principal trigger of the landslides, mostly earth slides class based on the Cruden and Varnes classification [34,35]. These landslide polygons were rasterized to a layer with 30-pixel meters obtained with 13,595 30-m pixels. We also randomly made a subset raster with 13,597 pixels with no landslides. Finally, we created a dataset with a binary variable called landslide presence by combining these two layers (presence or nonpresence of landslide).

#### 2.2.2. Susceptibility Map

From the inventory of landslides and the variables that have been considered as conditioning factors and triggers when predicting landslides, it has been possible to obtain a map based on machine learning technologies [24]. Bruzón and collaborators [24] made a susceptibility map using machine learning models and remote sensing resources. The variable to predict in the susceptibility model was the presence of the landslides identified, in the Section 2.2.1 of this work, from the landslide inventory. The explanatory variables are those shown, as follows, in Table 1.

For susceptibility analysis, thirteen variables were selected based on the variables most used in the literature to develop susceptibility evaluations of landslides [32,36,37]. Assigning the variables for this study followed two conditions: the information’s availability and the a priori effect these variables have on landslides (Bruzón and collaborators).

Shuttle radar topography mission (SRTM) provides a near-global scale digital elevation model using radar interferometry. NASA JPL delivers this product with a resolution of 1 arc-second (approximately 30 m) [38]. Using ArcGIS software tools, we calculated the slope, aspect, drainage network, and standard terrain curvature from the STRM elevation model. The aspect was categorized into nine classes: North-East, East-North, East-South, South-East, South-West, West-South, West-North, North-West, Flat.

With the help of the daily surface weather and climatological summaries (Daymet), we could obtain the climatic variables for the landslide susceptibility analysis. Daymet is a dataset with estimated daily meteorological parameters for North America, Hawaii, and Puerto Rico, with a resolution of 1 km. We measured the average annual precipitation between 1 January and 31 December 2012 for the susceptibility analysis through a script in Google Earth Engine [39].

Susceptibility assessment is very sensitive to geological variables. This is because the main spatial geological sources are geological maps. However, geological maps are not elaborated for the specific purposes of landslide studies, and, for that reason, we categorized the geological map to reinforce the relationship between geology categories and landslides. The geological information was provided by the National Institute of Geography and Informatics Statistics of Mexico (INEGI). The geological INEGI sources have data from the origin, classification, and the age of the rocks, faults, fractures, volcanoes, mines, etc., at a scale of 1:250,000 [40]. From the vectorial geological data from INEGI, we developed the lithotechnical categorical variable and the variables made from the lineaments (see Table 1). Geological information from the susceptibility analysis of the study have 47 different geological units. Those 47 units were reclassified into broader units according to lithological criteria, genetic process (igneous and sedimentary), and, among them, the geotechnical processes suffered (cohesion), which are potentially related to landslide susceptibility [41,42,43,44]. As is explained by Bruzón and collaborators [24], the multilevel information was reclassified and guided by expert decision, obtaining a clusterization of seven categories: (1) sedimentary materials (sands, silts and/or conglomerates), (2) volcanic–sedimentary igneous materials (tuffs, breaches, volcanoclastic), (3) volcanic igneous materials (andesites, basalts, dacites), (4) plutonic igneous materials (granites, granodiorites, syenites), (5) metamorphic materials (quartzites), (6) sedimentary materials (limestones), and (7) sedimentary materials (gypsum and carbonates). Sedimentary lithologies, such as sands, silts, and conglomerates, are the materials most susceptible to sliding. At the same time, the plutonic igneous rocks (granites, granodiorites, syenites), metamorphic lithologies (quartzites) and chemical sedimentary rocks (limestones and carbonates) are the materials least susceptible to sliding. We also calculated the Euclidean distance to lineaments and the lineament density with the help of ArcGIS software for the landslide susceptibility analysis (for more details, see Bruzón et al. [24]).

Anthropic variables for the susceptibility analysis (distance and density of road infrastructure) were obtained by the INEGI roads vector data. The road infrastructure vector data obtained from the INEGI dataset were used to calculate, with the help of the ArcGIS software, the two anthropic variables for the susceptibility analysis: distance to road infrastructure and density of road infrastructure. As it was explained in Bruzon and collaborators [24], infrastructure is related to landslides because of their destabilizing upper slopes through slope cutting, accumulating surface water, and hydrological pattern changes [45], overall, in the poorly built roads that are in the State of Guerrero [46].

Vegetation variables to consider in the susceptibility analysis were the normalized difference vegetation index (NDVI) and the landcover. The first mentioned was obtained by the Landsat 8 program, and the latter vegetation variable was downloaded and provided by the Copernicus Global Land Service [47]. The NDVI images from the Landsat 8 Collection 1Tier 1 [48] have a periodicity of eight days. Therefore, we used the average of the NDVI from summer (rainy season) of 2013. We used the vegetation variables in the susceptibility analysis because the lower the vegetation, the higher the landslide susceptibility [24].

For more details about the nature of the variables for the landslide susceptibility analysis, the previous work made by Bruzón and collaborators [24] can be consulted.

The methodology used in the susceptibility analysis was split into three phases. Firstly, we established the variables for the study from the information sources to build the raw dataset. Secondly, we developed an exploratory data analysis, in which two datasets were generated. One of these aforementioned datasets was used for training and testing, and the other was used to obtain the susceptibility map. Secondly, we made an automatic model selection based on the results to train and test data. Finally, we developed a probabilistic prediction of susceptibility to create a landslide susceptibility map in the entire study area with the best model identified (extra tree classifier model). The open-source Pycaret library [49] was used in the model generation phase to be able to make a comparison of different machine learning models. PyCaret is a Python open source machine learning library designed to easily complete standard tasks in a machine learning project. Pycaret allows models to be evaluated, compared, and tuned on a given dataset with just a few lines of code.

The accuracy of the susceptibility model is 0.977. Bruzón et al. [24] tested 16 machine learning models and compared them to obtain the best (best accuracy) model (the model developed through extra trees classifier). With the help of this model, then, we created a susceptibility map (probabilistic prediction of landslide susceptibility map) that we present and use in the present paper to take the first attempt at mapping danger.

The susceptibility is categorized into four levels (from areas with a very low probability of landslides to regions with a very high chance of landslides) using the natural cuts method (Jenks) [50]. To delve into the susceptibility analysis approach in the same study area that is the focus of this work, see the research of Bruzón and collaborators [24].

### 2.3. Ecological Values

Ecological values include biodiversity, conservation status, and habitat fragmentation level. With the help of GIS software, we have resampled and reprojected all the layers to the same pixel size and projection (ETRS 1989 UTM Zone 30 N). 

#### 2.3.1. Biodiversity

Net primary productivity (NPP) is gross primary productivity minus the rate of energy loss to metabolism and maintenance, i.e., NPP is the rate at which energy is stored as biomass by plants or other primary producers and made available to consumers in the ecosystem. It serves as an indicator for total species richness (biodiversity) in areas with a high level of net primary production, since these areas have more resources to host life among competing species, both animals and plants [51].

Using remote sensing data, NPP can be assessed through photosynthesis values. All this is possible thanks to MODIS (moderate resolution imaging spectroradiometer), an instrument carried by the Terra satellite that measures the physical properties of the atmosphere and the biological properties of the oceans and soils with 36 spectral bands, 21 between 0.4–3.0 µm and 15 between 3–14.5 µm [52].

In this case, MOD17A2H version 6 Gross Primary Productivity (GPP) has been chosen, a NASA EOSIDIS product that accumulates eight days with a resolution of 500 m. It includes data from the GPP and PSN (net photosynthesis) and quality control from PSN [53].

To do this, the MODIS team has used satellite derived fraction of photosynthetically active radiation (FPAR) (from MOD15) and independent estimates of PAR (photosynthetically active radiation) and other surface climate fields (from DAO data, CMMS/NASA), the maintenance and growth respiration terms that are subtracted from the GPP, to arrive at the annual NPP that has subsequently been estimated. The components of maintenance respiration (MR) and growth respiration (RG), which link the daily biomass and the yearly growth of plant tissues with the estimates of the leaf area index obtained by satellite, are based on the allometric relationships. The latest mentioned has been developed through an extensive bibliographic review and incorporating the same parameters as those used in the process model of the BIOME-BGC ecosystem [54].

The assigned values for the ecological value evaluation from the NPP variable were selected by the method of natural breaks.

#### 2.3.2. Conservation Status

Data from protected natural areas (PNA) and naturalness index (NI) have been used. Protected and natural regions could have higher ecological values than not fully protected and artificial ones. Thereby, the first mentioned will be more vulnerable (ecological vulnerability) than the second ones.

Protected natural areas are considered to be the “clearly defined, recognized, dedicated and managed geographic space, through legal resources or other types of effective resources to achieve the long-term conservation of nature and its ecosystem services and its associated cultural values” (http://www.unep-wcmc.org, accessed on: 11 September 2021). Through Protected Planet, the UNEP-WCMC (UN Environment Program World Conservation Monitoring Center) and IUCN (International Union for Conservation of Nature) make available to the user a reliable database of protected areas (terrestrial and marine) updated monthly, the World Database on Protected Areas (WDPA). It compiles information from almost 500 sources obtained from governments, communities and collaborating partners [55].

We define the naturalness of ecosystems (naturalness index) as an area that has not been made or influenced by humans [56]. We obtain a naturalness index through the landscape mosaic (LM) tool included in the Guidos Toolbox (GTB) [57] and by using as land cover continuous base the land cover maps provided by the Copernicus Global Land Service, which represent spatial information on different types (classes) of physical coverage of the Earth’s surface, e.g., forests, grasslands, croplands, lakes, wetlands, urban. This land cover map is captured from the PROVA-V sensor from the European Space Agency (ESA), with 100 m and an annual temporal resolution. From the present work, the authors downloaded the land cover map from 2015. This map was the input layer for the LM tool. LM tool catalogued land in a tripolar classification of a location for the relative contributions of agriculture, natural and developed land covers. The tripolar classification scheme split the space into 19 mosaic classes [58].

To transform the categorical classification of LM into a discrete naturalness index, we changed the LM categories into an index based on the thresholds defined in the LM method. As a result, we obtained a variable where 0 would correspond to the most artificial ecosystem types, and four corresponds to the most natural ecosystems. Table 2 shows the values assigned to each LM category.

#### 2.3.3. Habitat Fragmentation

Fragmentation is defined as transforming a large habitat into others isolated from each other and with a smaller surface, forming a matrix of habitats different from the original one [59]. Habitat fragmentation is considered a critical factor for the loss of biodiversity. Ecosystems with the least degree of fragmentation are considered the most vulnerable, as they are intact.

MSPA (morphological spatial pattern analysis) is a spatial analysis tool that detects and describes the morphometric characteristics of objects in digital images included in GTB [57]. This tool, called MSPA, belongs to the Forest project of the European Commission’s Joint Research Center. The code follows the analysis of morphological patterns, and the algorithm divides the foreground area of a binary image into seven distinguished MSPA classes (see Table 3): Core, Islet, Perforation, Edge, Loop, Bridge, and Branch [9]. According to this approach for developing a fragmentation map with the help of the MSPA tool, we used as input the already mentioned land cover map provided from the PROVA-V sensor, downloaded from the Copernicus Global Land Service (https://land.copernicus.eu/, accessed on: 11 September 2021). A habitat fragmentation (HF) index was made by weighting each MSPA class corresponding to its potential biodiversity conservation impact. The weights are shown in Table 4 and are taken based on a bibliography that reported conclusions about close relations between spatial coherence and resilience [9,60,61]. The higher vulnerability was assigned to less fragmented areas, i.e., core zones and those related to forest patches.

#### 2.3.4. Ecological Values Evaluation

The criteria to obtain categories for ecological values is shown in Table 4. In the end, we obtain four ecological values categories from the round-up mean of the three factors (biodiversity, conservation status and habitat fragmentation), where one means low, two moderate, three high and, finally, four implies very high ecological values. 

### 2.4. Ecological Regeneration Delay

The regeneration of ecological values will be closely related to the soil erosion potential because landslide events’ severity makes it impossible for the vegetation to develop adaptation strategies. Soil erosion potential is the inherent soil capability to avoid rainfall erosion. Our approach is based on four factors: (1) slope factor, (2) level of protection, (3) rainfall erosivity factor and (4) soil erodibility. 

(1) The slope factor: the slope was made with the help of GIS software from an SRTM, i.e., a near global scale digital elevation model, using radar interferometry. NASA JPL provides this product with a resolution of 1 arc-second (approximately 30 m) [38]. Table 5 shows the categorization of the slope to obtain the slope factor. 

(2) Level of protection: our approach divided the continuous study area into two categories: their level of protection being fully protected and nonfully protected areas, such as we have explained in Section 2.3.2, conservation status.

(3) Rainfall erosivity factor: the Global Rainfall Erosivity Database (GloREDa) is a database of the Joint Research Center (European Commission) that serves as input for the R Factor model of the universal soil loss equation (USLE) calculation. USLE is the method to be modified and then used to calculate the soil erosion risk estimate. This map has been obtained through a set of pluvial erosivity and rainfall data from different precipitation stations around the world, with a temporal resolution of 1 to 60 min and a pixel size of around 1 km, in addition, a Gaussian regression model to obtain the R Factor map by interpolating the erosivity values of the precipitations of the individual stations [62]. Table 6 shows the rainfall erosivity factor categories.

(4) Soil erodibility: The Soil Erosion Data Set, Scale 1:250,000 Series I Continuous National of the INEGI [31] is a data layer that contains eroded areas with types, shapes and degrees of erosion, in addition to data on environmental conditions and physical characteristics of the soil. Table 7 shows the rainfall erodibility factor categories.

Finally, the soil erosion potential, closely related to the ecological regeneration delay, is calculated through the aggregation of the four factors described below, carried out by a cross tabulation procedure shown in Table 8.

The ecological regeneration delay (ERD) indicates how long after a landslide the pre landslide conditions could be expected to be re-established. Hence, the greater the ecological regeneration delay values, the higher the landslide vulnerability.

### 2.5. Landslide Ecological Vulnerability Assessment

Table 9 shows the criteria followed to obtain the integration of ecological values and the results of the ecological regeneration delay. By crossing the ecological value map with the resulting ecological regeneration delay map, we obtained the landslide ecological vulnerability final map with four categories: low, moderate, high, and very high landslide ecological vulnerability. We considered that higher ecological values and a larger regeneration delay imply greater landslide ecological vulnerability values on the map.

### 2.6. Landslide Socio-Economic Vulnerability Assessment

Landslide socio-economic vulnerability approach considers the integration of the three factors: marginalization index, population density and building density.

#### 2.6.1. Marginalization Index

Marginalization is the expression of the underdevelopment degree of a specific area. It is related to socio-economic backwardness and is determined by living conditions, contributing to inequalities in people’s realities. Therefore, zones with high values of marginalization index could be more vulnerable than others, with low values.

The marginalization index makes it possible to differentiate the deficiencies suffered by the population according to the global impact [63]. The National Commission for the Knowledge and Use of Biodiversity (CONABIO) (https://www.gob.mx/conabio, accessed on: 11 September 2021) provide the marginalization index. CONABIO calculates the marginalization index because they have information from the Population and Housing Census to develop some socio-economic indexes in Mexico. The indicators taken into account to calculate the marginalization index are literacy status, educational level, housing, drainage, toilet, availability of energy and water, bedroom, the material of the floor of the house, locality, and size locality, employed population and income from work.

Marginalization index is downloaded free from the INEGI (CONABIO) as GIS vectorial layer with a spatial scale of 1:250,000.

#### 2.6.2. Population Density

Population density is the number of people living per km^2^; it must be considered when calculating vulnerability. A higher density implies greater vulnerability since there is more probability of damage to human beings and property and services. For instance, it is important to highlight those regions with very dense populations, which are more difficult to evacuate and care for during emergencies [64].

The Global Human Settlement Layer (GHSL) project offers global spatial data about the human presence on the planet over time. This is in the structure of built up, population density and settlement. This information is produced with evidence based analytics using new spatial data mining technologies (CITA). Regarding population density, the GHS-POP product is a spatial raster dataset representing the distribution of population, expressed as the number of people per cell; estimates for target years 2015 provided by CIESIN GPWv4.10 were disaggregated from census or administrative units to grid cells.

#### 2.6.3. Building Density

Building density is defined as the urban fabric space that exists in a specific area. Therefore, zones with high building density could be more vulnerable because it is expectable that high building density may be associated with high population density. Residential building densities give an idea of a region’s population disaggregation [65]. As Cutter et al. [64] affirm, the density of residential construction affects potential losses and recovery. We obtained the building density as a layer from GHS-S2Net grid derived from Sentinel-2 global image composite for the reference year 2018 using Convolutional Neural Networks. It builds on a new deep learning framework for the pixel-wise large scale classification of built-up areas at 10 m of resolution. 

#### 2.6.4. Integration of the Three Socio-Economic Vulnerability Factors

Higher values of these three factors imply greater landslide socio-economic vulnerability, thereby, to integrate them, Equation (1) was followed.

LSEVnum = MI + PD + BD,(1)

LSEV numerical is the landslide socio-economic vulnerability, MI is the marginalization index, PD is the population density, and BD is the building density. 

Once the map is calculated from the LSEVnum, we obtain the LSEVcat or LSEV categorical by applying natural breaks to the LSEVnum. Thus, we obtain the LSEV in 4 categories: one implies low, two moderate, three high, and four means very high socio-economic vulnerability.

### 2.7. Integration of Vulnerability Components

Once the different components of our vulnerability assessment were obtained, the final integration was undertaken using a qualitative cross-tabulation, prioritizing the most valuable element (see Table 10).

### 2.8. First Attempt Risk Assessment through the Integration of Vulnerability and Susceptibility Map

Finally, we perform an assessment of the risk by crossing our susceptibility map with the vulnerability one, following a bivariate pattern in GIS software.

## 3. Results

### 3.1. Susceptibility Map

**Figure 2:**
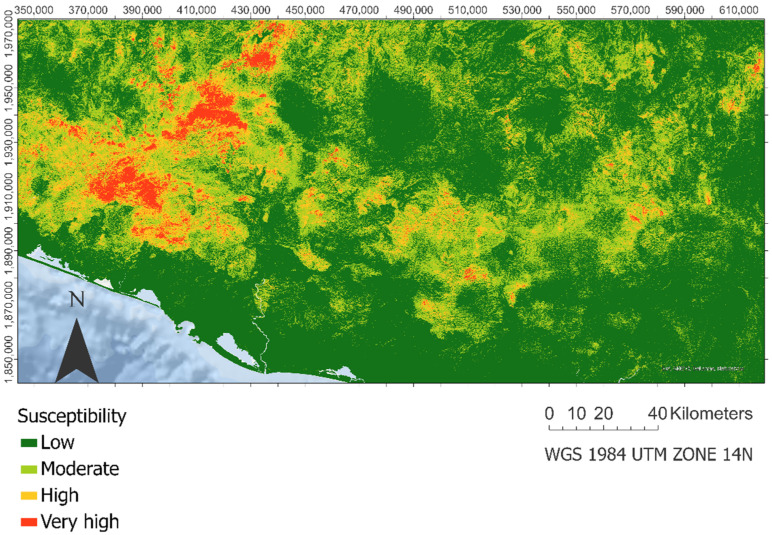
Landslide susceptibility map based on the probabilistic prediction of the extra trees model.

Figure 2 shows the result of the landslide susceptibility map of the entire study area [24]. It is observed how the areas with the highest probability of landslides are concentrated in the west of the study area and run through the Sierra Madre del Sur. Furthermore, the areas of greatest susceptibility coincide with those areas where the density of lineaments and accumulated precipitation are high, on lithologies susceptible to landslides. Finally, in the areas closest to the ocean and flat regions, the susceptibility is very low.

### 3.2. Landslide Ecological Vulnerability Assessment

**Figure 3:**
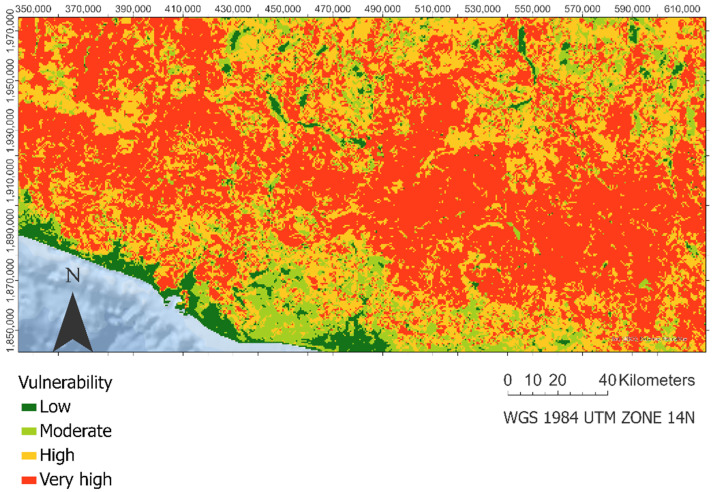
Landslide ecological vulnerability.

Figure 3 shows the spatial distribution of ecological vulnerability measures by integrating the ecological values and the ecological regeneration delay. We observed a dispersed and wide distribution of high and very high vulnerability areas along with the Sierra Madre del Sur mountain range.

### 3.3. Landslide Socio-Economic Vulnerability Assessment

**Figure 4:**
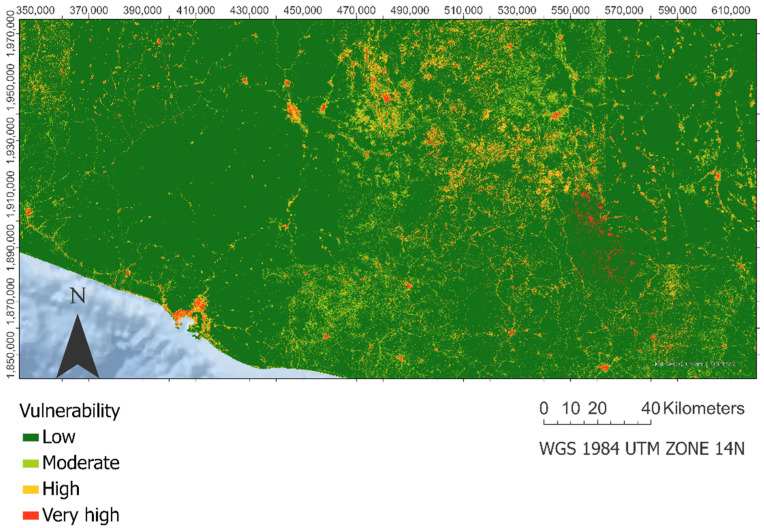
Landslides socio-economic vulnerability.

Conversely, in Figure 4, it can be seen how, in the spatial distribution of socio-economic vulnerability, the low category is predominant, observing clustered areas of high and very high vulnerability around urban areas and on roads of the municipalities with higher rates of marginality.

### 3.4. Integration of Vulnerability Components

**Figure 5:**
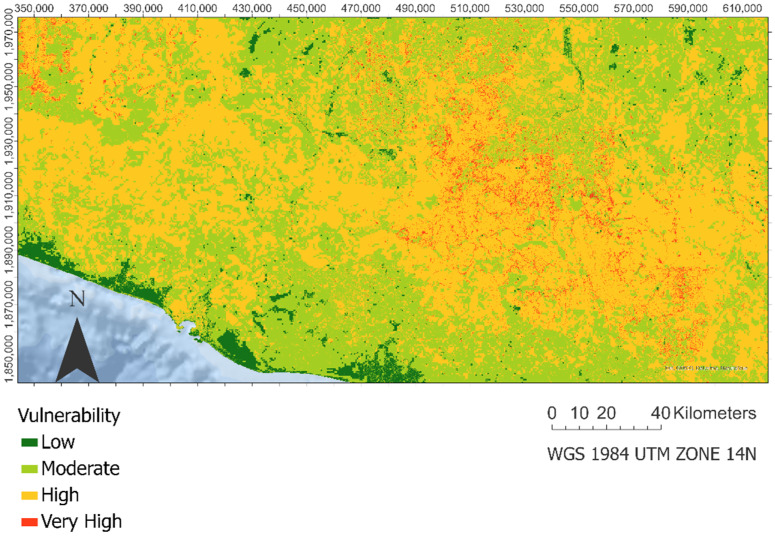
Landslide vulnerability integration. This includes the estimation of ecological vulnerability and socio-economic vulnerability.

The differences in the spatial distribution of these two vulnerabilities will generate a vulnerability map (Figure 5), in which the predominant category is vulnerability to significant landslides throughout the study area, and areas of low and moderate vulnerability are found in the nonurban zones near the coast and some of the valleys north of the Sierra Madre del Sur mountain range. Additionally, the map shows concentrated areas of very high vulnerability to landslides in the vicinity of small urban centers and their road network in the east center of the study area.

### 3.5. Risk Assessment

**Figure 6:**
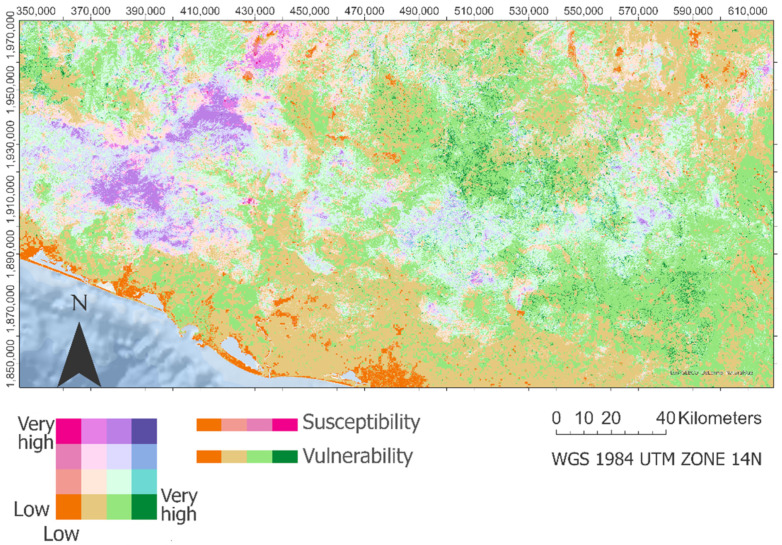
Landslide risk assessment. Includes the estimation of landslide susceptibility and landslide vulnerability.

Figure 6 shows the risk distribution from aggregating the susceptibility and vulnerability maps. From this aggregation, we could see how, in the coastal areas and the north west part of our study area, there is a concentration of zones with low risk, i.e., the lowest vulnerability and susceptibility. It is important to highlight how, also in the coastal areas, we find some sites or pixels in our risk map in which the vulnerability is high, and the susceptibility is low. These areas with high vulnerability and low susceptibility in the coastal zone coincide with the urban areas (e.g., Acapulco) (see Figure 6). In the mountainous zone east of our study area, especially at the foot of the mountains, we can also appreciate that the vulnerability is high, and the susceptibility is low.

From Figure 6, we can also notice how the areas with high risk (high vulnerability and high susceptibility) are generally concentrated in the mountainous zones, overall, in the west.

Finally, it seems important to mention there is an area in the north west part of our study zone in which we can appreciate high susceptibility in addition to low vulnerability. The latter mentioned is because, in this zone, there are low ecological values and low socio-economic values. Still, the physical characteristics of the terrain made that the probability of landslide occurrence is high.

**Figure 7:**
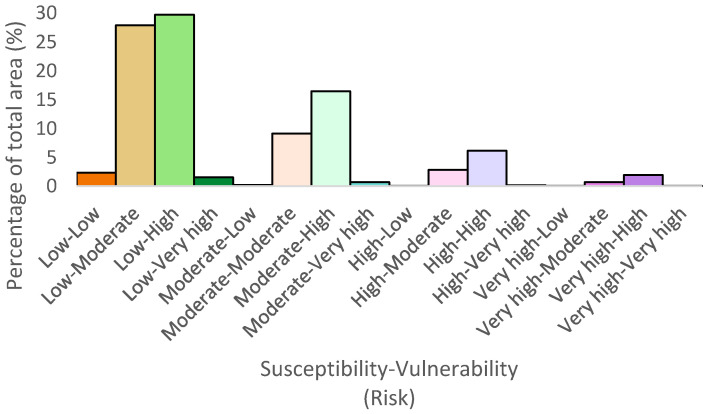
Area percentage of each risk category.

Figure 7 shows the great difference between the frequency of vulnerability and susceptibility pixels in the study area. This fact could be noted in the low–moderate category and the low–high one, both the most representative categories in our approach. Then, we could find how class moderate–high (moderate susceptibility and high susceptibility) presents more than 15% of the total area, being one of the most representative types from our study.

## 4. Discussion

Assessing current landslide vulnerability is very important for improving landslide prevention and landslide risk assessment tools. This paper presents a first attempt to evaluate risk integrating susceptibility made by ML methods, and vulnerability to landslide considering the ecological values and the socio-economic values in the state of Guerrero. Identifying susceptibility to suffering a landslide event is an important tool for planning decision making [23,66].

Our evaluation was based on the best existing spatial databases to cover this area. We have used free products from spatial databases, and some derived from remote sensing. Nowadays, remote sensing products provide a realistic alternative to obtain land surface information across broad regions and over longer periods at a low cost [67]. This spatial approach to assess the risk by the vulnerability and the landslide susceptibility is the most proper way to challenge this type of disaster [10,11].

The high landslide risk areas within our study zone are found in the mountain zones, where the high susceptibility and vulnerability values are great. In those mountainous areas, the landslide occurrence is high because the predisposing factors are propitious (high accumulation of precipitation and density of lineaments on lithologies susceptible to landslide). These results match the results reported in other studies that have assessed landslide risk [14,68,69]. In addition, the mountainous zones also have great ecological values. Furthermore, those zones concentrate the highest marginalization values from the entire study area.

In contrast, our study’s low landslide risk areas are found in zones where the susceptibility is low, as well as the vulnerability. Regarding the low susceptibility, these areas are centered in geolithologies prone to slides, with gentle slope values. These patterns of susceptibility are also shown in Menggenang and Samanta [70]. Regarding the vulnerability, in those low-risk areas, the ecological values are modest, in addition to low values from the marginalization index due to being the zones with the highest percentage of employment based on the third economic sector trade and tourism [71]. However, on this occasion, there are, in our map (Figure 6), areas with high vulnerability in zones with great density of population values around urban areas [9].

On the one hand, the novelty of our approach is that we consider the assessment of the vulnerability to landslide and not only the susceptibility. Nowadays, many works focus only on the susceptibility assessment (made by classical statistics or by machine learning and deep learning methods) (see for instance: [12,37,72,73,74]). On the other hand, we consider the ecological values and not only the socio-economic, which is more frequent to see in the existing literature (see for instance: [65,75]). The assessment of the ecological values within vulnerability evaluation is important, because they are related to socio-economic values. This is due to the fact that ecosystem services reinforce all aspects of human well-being—from basic livelihoods to moderate success, and sustainable development [76], promoting the achievement of sustainable development goals (SDGs). Overall, those communities are those whose livelihoods depend mainly on ecosystem goods and services (e.g., timber, mining, agriculture) [77].

As our results show, assessing the ecological vulnerability in addition to the socio-economic one is essential. We have areas with high susceptibility in which the socio-economic vulnerability value is low, but the ecological value is high. Therefore, in these areas where the probability of landslide occurrence is great, the expected ecological losses would increase. If we only consider the socio-economic vulnerability values, the vulnerability values will be low; thereby, the risk value would be low. From our results, it can be noted how the ecological vulnerability and the socio-economic are distributed very differently. The ecological values are important because they are indirectly related to the level of life and the socio-economic values in a zone. In areas with high vulnerability to landslides, mainly caused by the fact that the density of population or the marginalization is great, the recovery after a landslide event would be lower and, thereby, the final losses greater because of those additional losses in ecosystem services provision or the biodiversity of an area (in definitively the ecological values).

Besides, the ideal evaluation of ecological vulnerability must take into account both the biotic and abiotic factors of the environment, as well as the relationship between them (ecosystem functionality) [78] as the inability of an ecosystem (structural and functional component) to tolerate temporal and spatial pressures [21]. This approach is followed in the present work (see Figure 1).

In this study, we focus on a first approach to estimating the risk of the environment to landslides. We focus on building a methodology with the available data sources that allow us to obtain a first risk map based on integrating the landslide susceptibility and vulnerability. However, given the lack of temporal data on past landslides frequency and magnitude, we had to analyze the hazard using only the susceptibility map as a proxy of hazard. This limitation matches other studies related to the landslide risk reported in the literature [79,80,81].

For future research, we consider the exploration of artificial intelligence and big data to assess the vulnerability to landslides. Here, we have explored a heuristic approach to evaluate the vulnerability, but we could examine the possibility of using logic diffuse, machine learning, deep learning methods for future works. Besides, we consider exploring the potential to analyze bank card payments and ATM cash withdrawals to map and quantify how people are impacted by and recover from a landslide disaster. In this sense, a big data approach could result in a better understanding of economic resilience and could be enriching to assess the risk of landslide events.

## 5. Conclusions

Landslides have been considered as one of the most critical natural hazards that present a serious threat to life and property in the world. Therefore, short term and long term solutions are mandatory to meet these overwhelming challenges. The landslide risk assessed by the integration of susceptibility and vulnerability map has recently become a manner of studying the sites prone to landslide events and managing these regions well. This work offers a methodology based on the susceptibility map, obtained in a previous study through AutoML modelling, and assessing the vulnerability to landslide (considering ecological and socio-economic vulnerability). Our study highlights the importance of integrating ecological values (ecological vulnerability) and socio-economic landslide vulnerability to assess the risk of landslide disasters. In this sense, our approach is a novel prototype for the first attempt of risk to landslide. We have used remote sensing and spatial databases that are free, machine learning technologies (for the susceptibility map) and ecological and socio-economic vulnerability. This work is focused on the Guerrero State in México, but it could be applied to every part of the Earth and the fact that the input data were free means it has high possibilities of adaptation. Thereby, the present method could be essential to manage developing countries prone to landslide disasters.

## Figures and Tables

**Table 1:** Explanatory variables to fit the model of susceptibility [24].

Variable	Source	Spatial Resolution (m)
Slope	SRTM	30
Aspect	SRTM	30
Distance to the drainage network	SRTM	30
Drainage network density	SRTM	30
Standard curvature of the Earth	SRTM	30
Cumulative annual precipitation	Daymet	1000
Litology	Geological chart	1:250,000
Distance to lineaments	Geological chart	30
Density of lineaments	Geological chart	30
Distance to road infrastructure	Communication routes	30
Density to road infrastructure	Communication routes	30
NDVI	Sensor Landsat 8	30
Land cover	Copernicus Global Land Service	100

**Table 2:** Values assigned to each LM category.

LM Category	Discrete Value
A	0
D	0
N	3
Ad	0
An	1
Dn	1
Da	0
Na	2
Nd	2
Adn	1
Dan	1
Nad	2
ad	0
an	1
dn	1
adn	1
NN	4
AA	0
DD	0

**Table 3:** Weights for the morphological spatial pattern analysis (MSPA) classes (Wj), taking into account the assessment of habitat fragmentation [9].

MSPA Categories	Definition	Wj
Perforation	Borders of nonforest islands within the forest matrix	1.3
Bridge	Pixels joining two forest patches	1.5
Core	Pixels within the forest matrix	2
Background	Nonforested areas	1
Islet	Forest islands outside the forest matrix	1.1
Branch	Forest corridor linked to a forest patch	1.2
Loop	Pixels joining the same forest patch	1.2
Edge	Borders of the forest matrix	1.3

**Table 4:** Criteria to obtain categories for ecological values. NPP means net primary production, PNA is the acronym of protected natural areas, NI means natural index, LM is landscape mosaic, HF is the abbreviation of habitat fragmentation, MSPA is the abbreviation of morphological spatial pattern analysis.

Factor	Variable Name	Variable Range	Assigned Values
Biodiversity	NPP	0–1,000,000 (g C)	Values between 1 and 4 following natural breaks
Conservation status	PNA	0–1	0 = 0
			1 = 4
	NI (from LM categories)	0–4	0 = 0
			1 = 1
			2 = 2
			3 = 3
			4 = 4
Habitat fragmentation	HF (from MSPA categories)	1–4	1–1.25 = 1
			1–1.50 = 2
			1–1.75 = 3
			1.75–2 = 4

**Table 5:** Assignation to each category of soil slope.

Discrete Variable	Definition	Slope Range in % Rise
1	Very gentle	<5
2	Gentle	5–15
3	Steep	>15–30
4	Very steep	>30

**Table 6:** Assignation to each category of rainfall erosivity factor.

Discrete Variable	Definition	Range
1	Low	<4
2	Moderate	4–8
3	High	>8

**Table 7:** Assignation to each category of rainfall erodibility factor.

Discrete variable	Definition	Range
1	Low	<3
2	Moderate	3–6
3	High	>6

**Table 8:** Vulnerability levels associated with the soil erosion potential (ecological regeneration delay).

Slope Factor	Level of Protection	Soil Erodibility and Rainfall Erosivity Factor		
		Low	Moderate	High
Very gentle	Fully protected	Low	Moderate	Moderate
Gentle	Fully protected	Low	Moderate	Moderate
Steep	Fully protected	Moderate	Moderate	High
Very steep	Fully protected	Moderate	High	High
Very gentle	Not fully protected	Low	Moderate	Moderate
Gentle	Not fully protected	Moderate	Moderate	High
Steep	Not fully protected	Moderate	High	High
Very steep	Not fully protected	Moderate	High	High

**Table 9:** Landslide ecological vulnerability resulting from the landslide ecological values and the ecological regeneration delay.

	Ecological Regeneration Delay			
Ecological Values	Low	Moderate	High	Very High
Low	Low	Low	Moderate	High
Moderate	Low	Moderate	High	Very high
High	Moderate	High	Very high	Very high

**Table 10:** Combination of ecological and socio-economic vulnerability assessment to obtain the integrated vulnerability to landslide.

Ecological Vulnerability	Socio-Economic Vulnerability			
	Low	Moderate	High	Very High
Low	Low	Moderate	Moderate	High
Moderate	Moderate	Moderate	High	High
High	Moderate	High	High	Very high
Very high	High	High	Very high	Very high
